# Supplementary material for: Dual-Mode Antibacterial Orthodontic Composite: Contact-Killing QACs and Sustained CHX Release via Large-Pore Mesoporous Silica Nanoparticles
Source: Int J Mol Sci. 2025 Jun 26;26(13):6172. doi: 10.3390/ijms26136172 (PMC12250240; doi:10.3390/ijms26136172)
Supplement: Supplementary file 1 [file ijms-26-06172-s001.zip › ijms-3594147-supplementary.pdf]

Supplemental Material

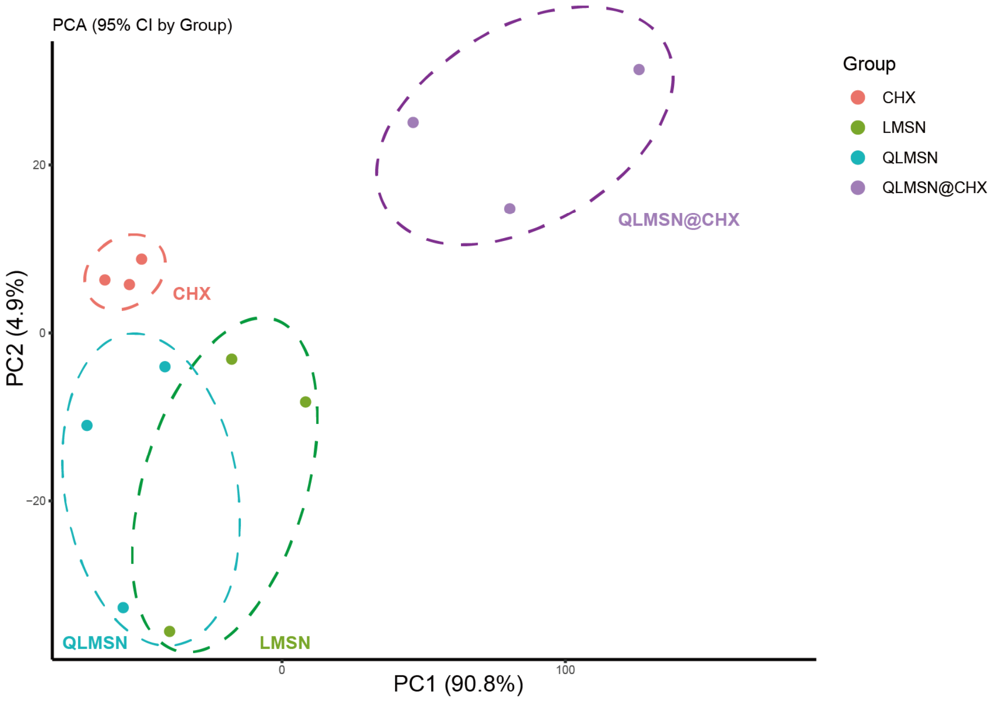

Figure S1. Principal component analysis (PCA) of different material groups.

PCA results revealed that the first two principal components (PC1 and PC2) collectively explained 95.7% of the total variance (PC1: 90.8%; PC2: 4.9%), indicating their effectiveness in capturing the core variation patterns of the data. Sample points were grouped by treatment (QLMSN, LMSN, QLMSN@CHX, GLMSN@CHX), with clear separation along PC1 (spanning 100 units on the x-axis), suggesting PC1 as the primary driver of inter-group differences. In contrast, PC2 showed minimal contribution, as groups largely overlapped (spanning only 40 units on the y-axis). Notably, QLMSN and LMSN were widely separated on PC1, implying significant differences between them, while CHX-treated groups (e.g., QLMSN@CHX) formed tighter clusters, possibly due to reduced variability. These findings highlight the pronounced impact of experimental treatments (e.g., CHX) or grouping conditions on data variation, with PC1 serving as the key dimension for subsequent analysis.

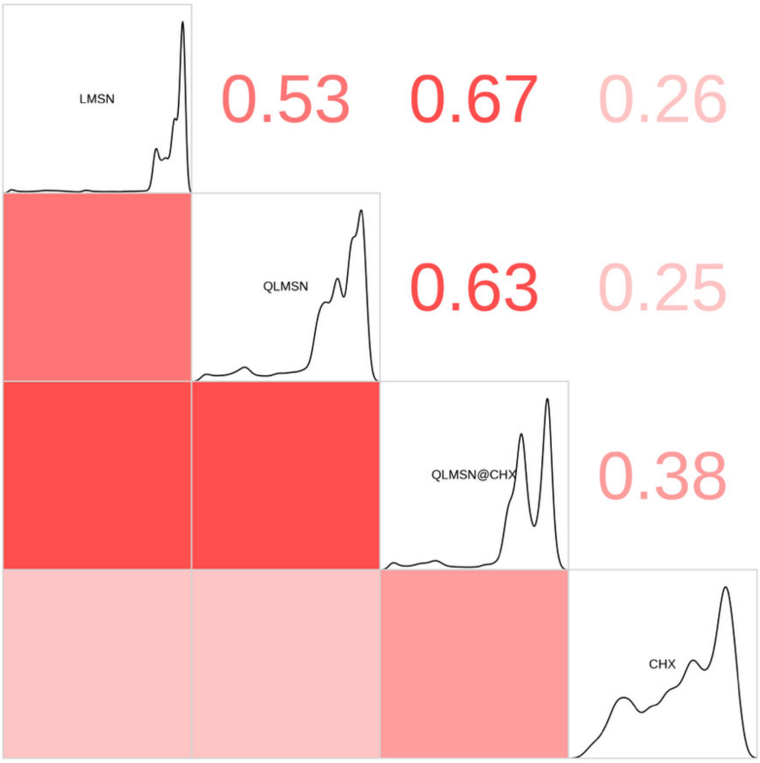

Figure S2. Parametric matrix analysis of different material systems

The matrix analysis reveals correlations/distances among experimental groups (LMSN, QLMSN, QLMSN@CHX, CHX). LMSN exhibits high intra-group heterogeneity (values: 0.53, 0.67, 0.26), while QLMSN shows moderate variability (0.63, 0.25) with limited samples. QLMSN@CHX presents a single value (0.38), possibly reflecting CHX-induced stabilization. CHX is listed without data, suggesting its role as a control. Notably, CHX treatment (QLMSN@CHX) may reduce dispersion (0.38 vs. QLMSN's wider range), aligning with tighter clustering in PCA—though further validation is needed.

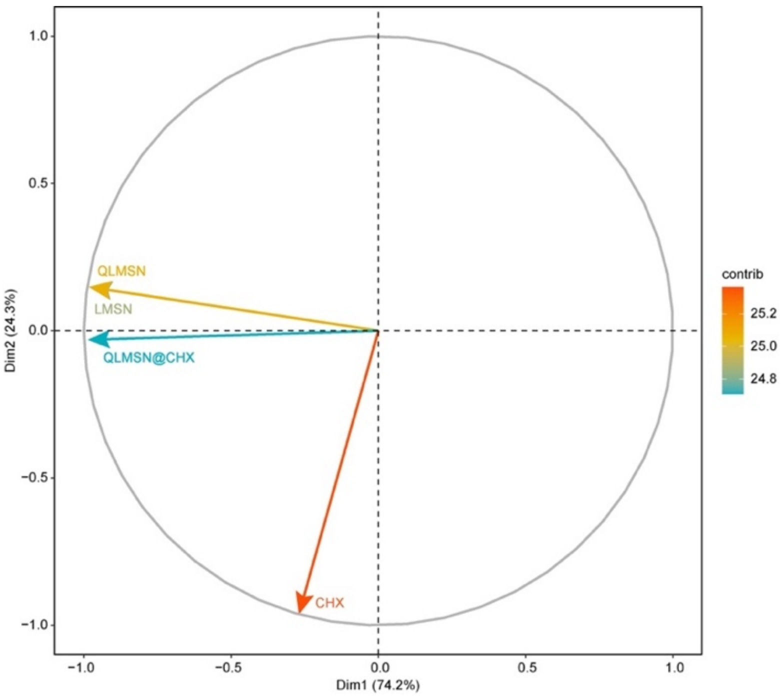

Figure S3. PCA Loadings Plot of different material systems

Principal component analysis (PCA) extracted four principal components (PCs), with the first two (PC1 + PC2) cumulatively explaining 98.48% of the total variance, indicating their effectiveness in capturing the core characteristics of the data. PC1 (74.21% variance) was dominated by "chlorhexidine loading," "quaternary ammonium functionalization," and "mesoporous silica" (with similar loading values, suggesting coordinated variation), reflecting the overall trend of material functionalization. PC2 (24.28% variance) was independently driven by "CHX" (loading: -0.977), representing a distinct variation direction unrelated to the other three variables. The variable contribution plot further confirmed strong positive correlation among the first three (small arrow angles), while CHX exhibited orthogonal variation (independent of others).

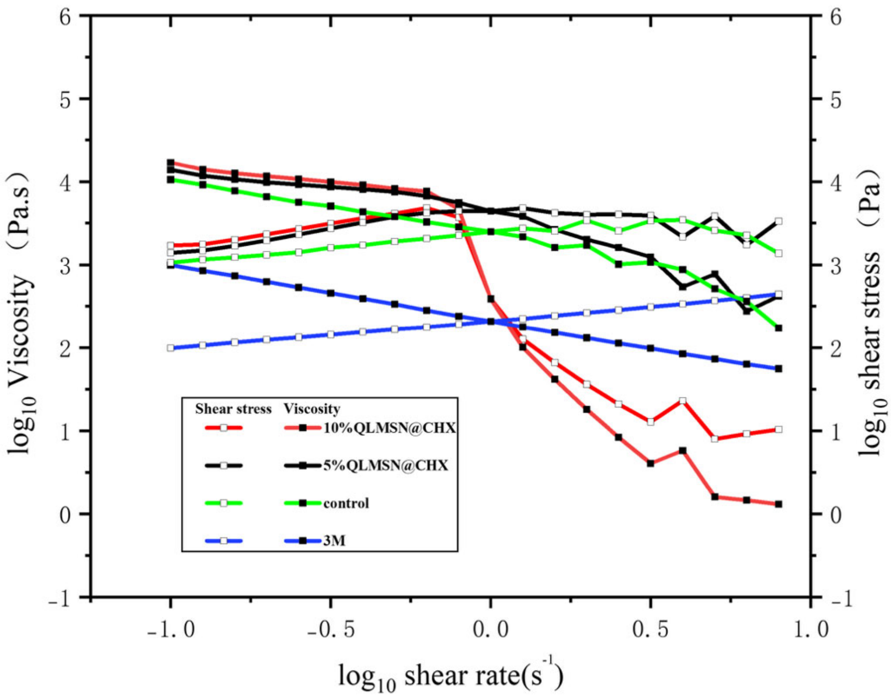

Figure S4. Rheological properties of composite materials

With increasing volume fraction of QLMSN@CHX fillers, the composite viscosity showed a significant enhancement. Double logarithmic plot showing the relationship between shear stress (Pa.s) and shear rate (s^{-1}). Notably, under identical conditions, the composite viscosity increased significantly with the rising mass fraction of silica particles[1].

The rheological characterization reveals that the resin system exhibits pronounced shear-thinning (pseudoplastic) behavior, as evidenced by a significant decrease in viscosity with increasing shear rate and nonlinear shear stress-shear rate profiles, which distinctly differs from the characteristics of shear-thickening, dilatant, or Bingham plastic fluids[2]. This pseudoplasticity likely originates from shear-induced disentanglement of polymer networks, orientation of nanofillers, or disruption of solvation layers. Importantly, the incorporation of QLMSN@CHX nanoparticles demonstrates a concentration-dependent influence on the rheological properties. At 5 wt% loading, the nanocomposite achieves optimal performance, maintaining appropriate viscosity with preserved shear-thinning behavior while avoiding the dramatic viscosity increase and processing challenges observed at 10 wt% due to nanoparticle aggregation. This threshold effect arises from nanoparticle-resin interactions: lower concentrations (2.5-5 wt%) enable homogeneous dispersion and moderate rheological

modification through surface effects, whereas higher loading (10 wt%) induces particle clustering that restricts polymer chain mobility through physical crosslinking. Notably, the 5 wt% formulation represents an ideal compromise, simultaneously ensuring efficient CHX loading, processability, and material stability - a critical combination for antimicrobial medical applications requiring both functional performance and manufacturing feasibility. These findings provide fundamental insights for designing nanoparticle-reinforced polymer systems with tailored rheological properties.

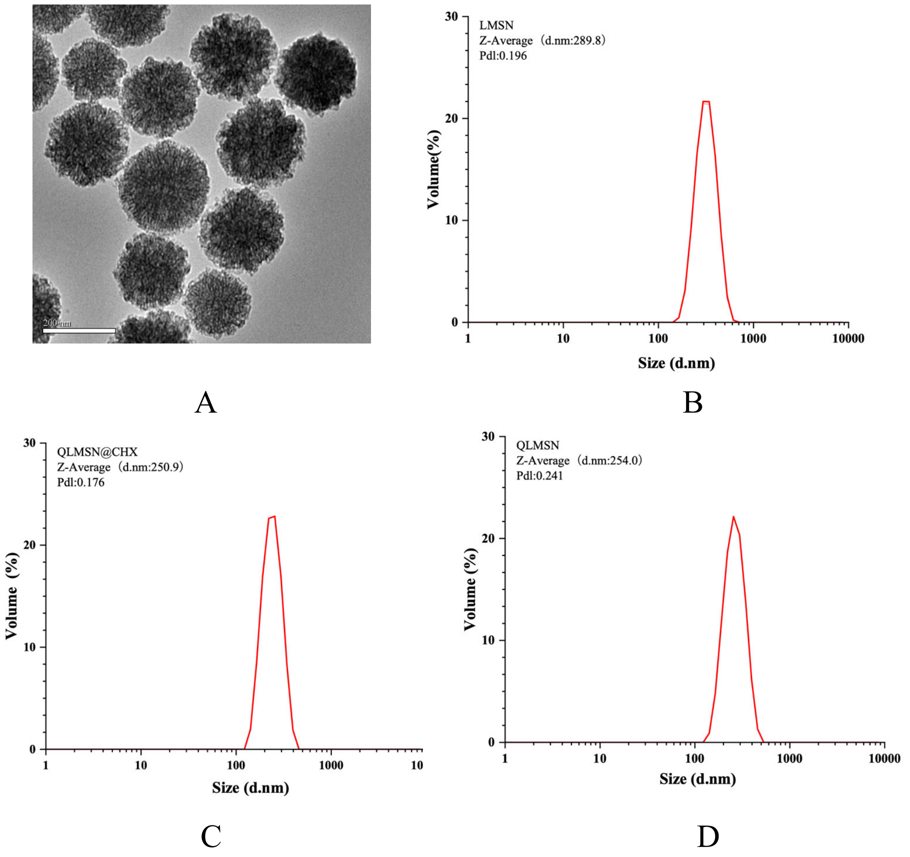

Figure S5. The DLS particle size distribution plots of LMSN, QLMSN, and QLMSN@CHX. (A) QLMSN@CHX (bar:200nm)

The nanoparticle size and zeta potential analyzer results showed that the diameters of LMSN, QLMSN, and QLMSN@CHX nanoparticles were approximately 200 nm. The increased particle size may be attributed to aggregation. Additionally, the PDI (polydispersity index) was around 0.2, and the particle size distribution exhibited a relatively narrow and normal

distribution, indicating excellent size uniformity of LMSN, QLMSN, and QLMSN@CHX nanoparticles.

TEM analysis in Figure S5.A reveals uniformly dispersed QLMSN@CHX nanoparticles with an average diameter of 200 nm, while DLS measurements showed larger particle sizes. This discrepancy may arise because: (1) DLS exhibits higher sensitivity toward larger particles and tends to amplify signals from aggregates; (2) the drying process during TEM sample preparation may dissociate loosely-bound aggregates present in solution, resulting in smaller observed particle sizes. Such differences are commonly observed in nanomaterial characterization - TEM provides more accurate information about primary particle morphology and size, whereas DLS better reflects the actual dispersion behavior in solution.

Interestingly, QLMSN and QLMSN@CHX showed smaller nanoparticle sizes compared to LMSN, which may be attributed to the electrostatic repulsion between positively charged quaternary ammonium and CHX groups that potentially reduces particle aggregation. Both TEM and DLS characterization results have been provided in the Supplementary Materials for comprehensive evaluation of the material's dispersion properties.

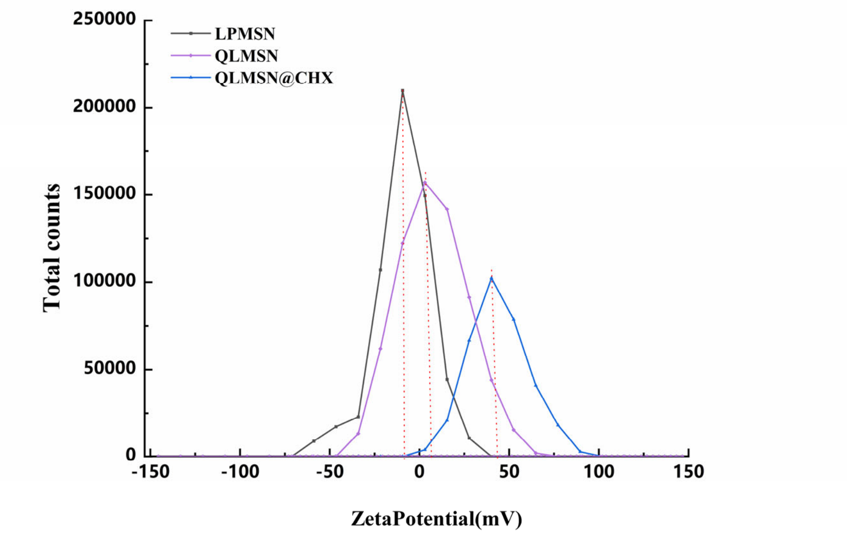

Figure S6. Zeta potential distribution of functionalized mesoporous silica nanoparticles

LPMSN: -35.2 ± 3.1 mV (native silanol negative charge), QLMSN: +48.6 ± 2.7 mV (quaternization-induced positive charge), QLMSN@CHX maintains positive potential (+32.1 ± 4.2 mV) but with reduced magnitude due to CHX masking.

Zeta potential measurements revealed that LMSN carried a negative charge, while QLMSN acquired a positive charge after quaternary ammonium functionalization. The charge remained unchanged after CHX loading.

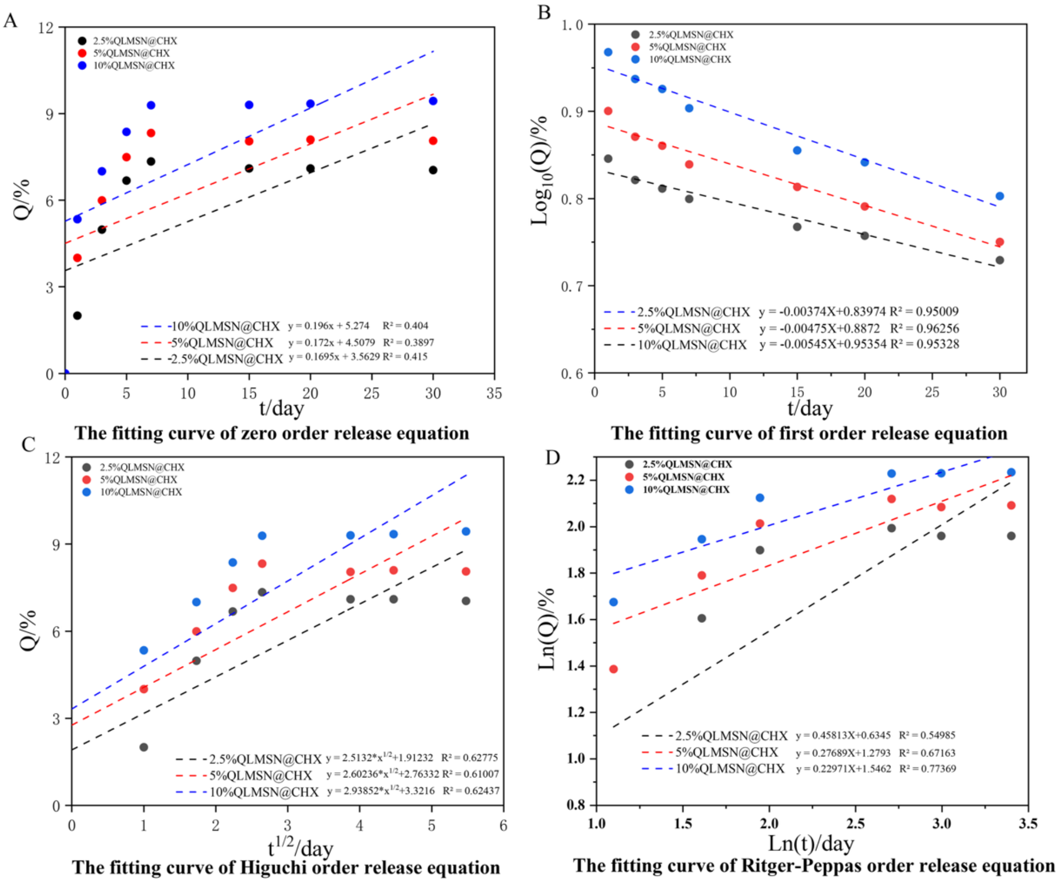

Figure S7. Fitting plots of CHX release kinetic models

According to the kinetic fitting results, the release behavior of CHX with different loadings from the QLMSN@CHX nanocomposite was analyzed using various kinetic models, including zero-order(Figure S7A), first-order(Figure S7B), Higuchi(Figure S7C), and Ritger-Peppas models(Figure S7D)[3-5]. By comparing the goodness-of-fit of these models, the first-order kinetic model (Figure S7B) demonstrated the best fitting performance, with a coefficient of determination (R^2) as high as 0.96256, significantly surpassing other models. This indicates that the release behavior of CHX closely follows first-order kinetics, where the release rate is proportional to the remaining amount of CHX to be released. Furthermore, the linear relationship between CHX release and time in the first-order kinetic plot further validates the applicability of this model. Therefore, based on the highest R^2 value and the physical

significance of the model, the first-order release kinetic model was selected as the optimal model to describe the release kinetics of CHX from QLMSN@CHX.

References

1. Zhang, X., Q. Zhang, X. Meng, Y. Ye, D. Feng, J. Xue, H. Wang, H. Huang, M. Wang, and J. Wang, Rheological and Mechanical Properties of Resin-Based Materials Applied in Dental Restorations. Polymers (Basel), 2021. **13**(17).
2. Li, Y., X. Wang, X. Lv, X. Wang, X. Wang, J. Cui, and M. Yan, Extractions and rheological properties of polysaccharide from okra pulp under mild conditions. International Journal of Biological Macromolecules, 2020. **148**: p. 510-517.
3. Alfei, S., B. Marengo, G. Zuccari, F. Turrini, and C. Domenicotti, Dendrimer Nanodevices and Gallic Acid as Novel Strategies to Fight Chemoresistance in Neuroblastoma Cells. Nanomaterials (Basel), 2020. **10**(6).
4. Mathematical models of drug release, in Strategies to Modify the Drug Release from Pharmaceutical Systems. 2015. p. 63-86.
5. Zuccari, G., S. Alfei, A. Zorzoli, D. Marimpietri, F. Turrini, S. Baldassari, L. Marchitto, and G. Caviglioli Increased Water-Solubility and Maintained Antioxidant Power of Resveratrol by Its Encapsulation in Vitamin E TPGS Micelles: A Potential Nutritional Supplement for Chronic Liver Disease. Pharmaceutics, 2021. **13**, DOI: 10.3390/pharmaceutics13081128.
